# Preexisting CD4+ T-Cell Immunity in Human Population to Avian Influenza H7N9 Virus: Whole Proteome-Wide Immunoinformatics Analyses

**DOI:** 10.1371/journal.pone.0091273

**Published:** 2014-03-07

**Authors:** Venkata R. Duvvuri, Bhargavi Duvvuri, Christilda Alice, Gillian E. Wu, Jonathan B. Gubbay, Jianhong Wu

**Affiliations:** 1 Centre for Disease Modelling, York Institute of Health Research, Toronto, Canada; 2 The Hospital for Sick Children, Toronto, Canada; 3 York University, Toronto, Canada; 4 Public Health Ontario, Toronto, Canada; 5 University of Toronto, Toronto, Canada; 6 Mount Sinai Hospital, Toronto, Canada; St. Jude Children's Research Hospital, United States of America

## Abstract

In 2013, a novel avian influenza H7N9 virus was identified in human in China. The antigenically distinct H7N9 surface glycoproteins raised concerns about lack of cross-protective neutralizing antibodies. Epitope-specific preexisting T-cell immunity was one of the protective mechanisms in pandemic 2009 H1N1 even in the absence of cross-protective antibodies. Hence, the assessment of preexisting CD4+ T-cell immunity to conserved epitopes shared between H7N9 and human influenza A viruses (IAV) is critical. A comparative whole proteome-wide immunoinformatics analysis was performed to predict the CD4+ T-cell epitopes that are commonly conserved within the proteome of H7N9 in reference to IAV subtypes (H1N1, H2N2, and H3N2). The CD4+ T-cell epitopes that are commonly conserved (∼556) were further screened against the Immune Epitope Database (IEDB) to validate their immunogenic potential. This analysis revealed that 45.5% (253 of 556) epitopes are experimentally proven to induce CD4+ T-cell memory responses. In addition, we also found that 23.3% of CD4+ T-cell epitopes have ≥90% of sequence homology with experimentally defined CD8+ T-cell epitopes. We also conducted the population coverage analysis across different ethnicities using commonly conserved CD4+ T-cell epitopes and corresponding HLA-DRB1 alleles. Interestingly, the indigenous populations from Canada, United States, Mexico and Australia exhibited low coverage (28.65% to 45.62%) when compared with other ethnicities (57.77% to 94.84%). In summary, the present analysis demonstrate an evidence on the likely presence of preexisting T-cell immunity in human population and also shed light to understand the potential risk of H7N9 virus among indigenous populations, given their high susceptibility during previous pandemic influenza events. This information is crucial for public health policy, in targeting priority groups for immunization programs.

## Introduction

On March 31, 2013, the China Center for Disease Control and Prevention identified a human infection by a novel avian influenza A virus (H7N9), one with multiple avian genetic reassortments [1], [2]. As of July 10, 2013, a total of 132 laboratory confirmed cases of human infection were reported, of which 43 (32.5%) were fatal. Epidemiological investigations indicated that most cases (77%) infected with H7N9 had contact with live animals including chickens. However, lack of family clusters and studies in animal models have highlighted the potential for human-to-human transmission of H7N9, with an added concern resulting from emerging mutants [3].

The avian specific genome and the antigenically distinct nature of H7N9 surface glycoproteins, led to the absence of protective neutralizing antibodies for H7N9 in the human population [4].The 2009 H1N1 pandemic witnessed the protective nature of preexisting CD4+ T-cell memory responses in human populations even in the absence of cross-reactive neutralizing antibodies [5]–[10]. Preexisting T-cell immunity directed towards epitopes that are highly conserved among seasonal influenza A(H1N1) and pandemic 2009 H1N1 subtypes was attributed to the milder severity of 2009 pandemic [5]–[14]. A human influenza challenge model by Wilkinson et al [9] observed a negative correlation between disease severity and preexisting CD4+ T-cell immunity directed towards conserved epitopes of influenza internal proteins with reduced viral loads. *In vitro* studies demonstrated the protective role of CD4+ T-cell reactivity against previously the unencountered avian influenza (H5N1) strain; this protection was shown to be due to the presence of commonly conserved and shared epitopes with seasonal influenza strains, H1N1 and H3N2 [15], [16].

Hence, preexisting CD4+ T-cell immunity can potentially limit the disease severity of H7N9 infection in antibody naïve population. Our study examines the likely presence of preexisting CD4+ T-cell immunity towards H7N9 in the human population, derived from previous exposures with human IAV subtypes (H1N1 1918–1976, seasonal H1N1 1977–2009, pandemic H1N1 2009–2013, H2N2 1957–1968, and seasonal H3N2 1968–2013). We conducted comparative whole proteome analyses and a large-scale immunoinformatics analyses to predict and identify the commonly conserved and shared CD4+ T-cell epitopes of H7N9 with human IAV subtype strains. Further, all the commonly conserved predicted epitopes among avian and human IAVs (henceforth referred as “commonly conserved”) were screened against the IEDB (Immune Epitope Database: contains experimentally identified epitope information) to validate their immunogenic potential. Next, we conducted population coverage analysis with the commonly conserved CD4+ T-cell epitopes in the context of Human leukocyte antigen (HLA) DRB1 alleles to understand the likely distribution of preexisting CD4+ T-cell immunity in different ethnic groups, and further discussed with previously reported influenza mortality/morbidity rates.

## Methods

### Methodological framework

**Figure 1:**
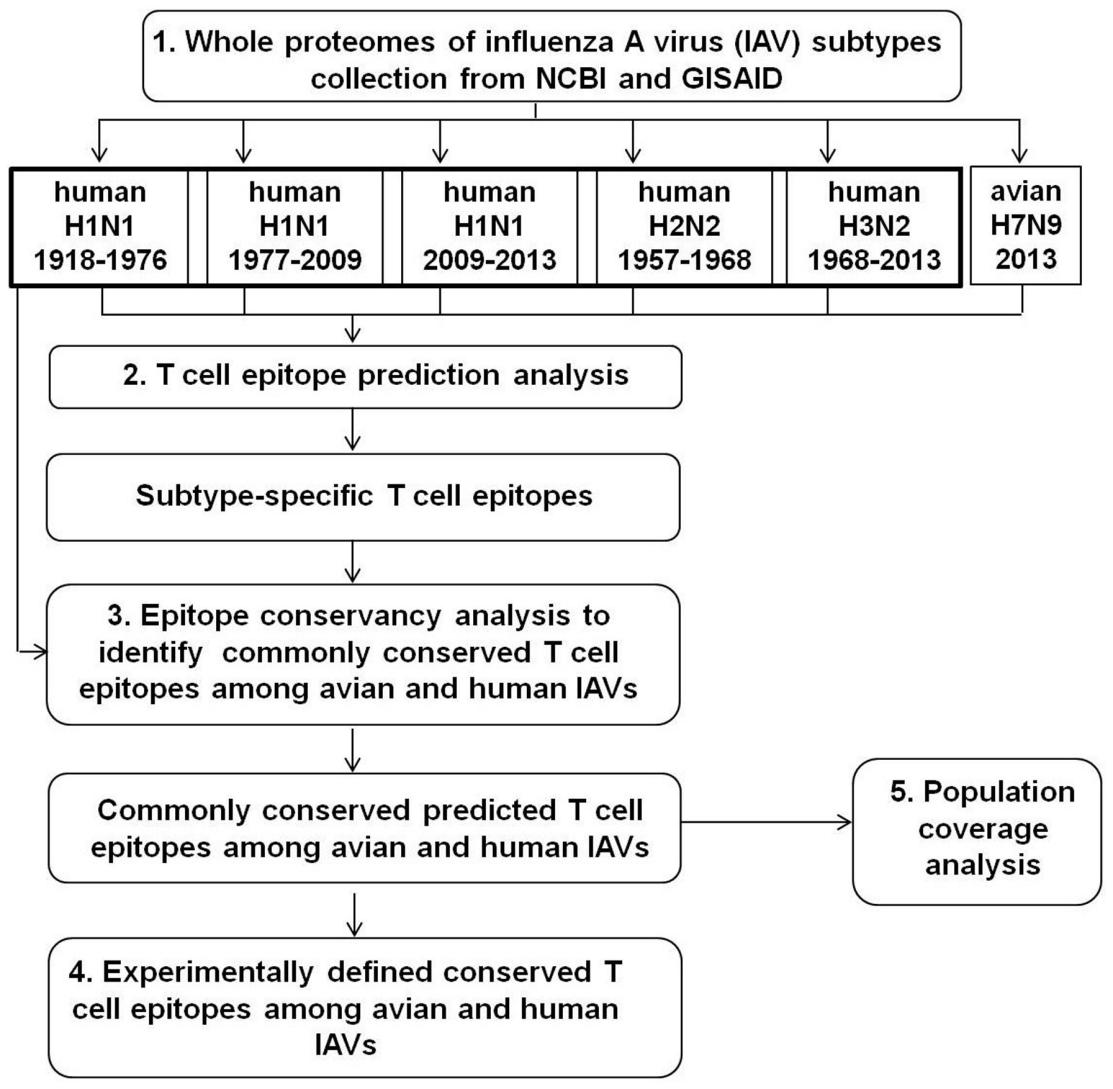
The methodological framework of the study.

Figure 1 presents an overview of the workflow of the current analyses based on earlier studies [6]–[8], [17]–[19]. In general, this framework consists five major steps: i) sequence collection, curation and analysis of IAV proteins from influenza genome databanks; ii) prediction of T-cell epitopes to fourteen HLA-DRB1 alleles using epitope prediction tools; iii) identification of commonly conserved predicted epitopes among avian and human IAVs using epitope conservancy tools; iv). experimental validation of commonly conserved predicted epitopes among avian and human IAVs based upon information in the IEDB; and v) population coverage analysis. This workflow was used to measure the preexisting CD4+ T-cell immunity in the human population against H7N9 virus and to identify a potential list of commonly conserved epitopes, including their population coverage.

### Sequence collection, curation and analysis

All eleven protein segments, namely polymerase B2 (PB2), polymerase B1 (PB1), PB1-F2, polymerase (PA), HA, nucleocapsid protein (NP), NA, matrix proteins 1 & 2 (M1 & M2), and nonstructural proteins 1 & 2 (NS1 & NS2) of H7N9 isolated from humans in China during 2013 were obtained from the Global Initiative on Sharing All Influenza Data (GISAID) Epiflu database (Table S1 in File S1). The proteomes of H7N9 (A/Shanghai/01/2013, A/Shanghai/2/2013, A/Anhui/1/2013, A/Hangzhou/1/2013); H1N1 1918 (A/Brevig Mission/1/1918, GISAID isolate id # EPI_ISL_1211); H2N2 (A/Singapore/1/57, GISAID isolate id # EPI_ISL_70062); H3N2 (the 2012–2013 vaccine strain: A/Victoria/361/2011, GISAID isolate id # EPI_ISL_101506); and Pandemic 2009 H1N1 (the 2012–2013 vaccine strain: A/California/07/2009, GISAID isolate id # EPI_ISL_31158) were used as reference strains to identify potential CD4+ T-cell epitopes. In order to estimate the comparative protein identity and conservancy of the H7N9 specific (predicted) epitopes of each protein, we considered the different subtypes of IAV protein sequences (particularly those isolated from humans) available in the GISAID and NCBI since 1918. A total of 101,310 protein sequences of different IAV subtypes were included in the analysis including 804 H1N1 1918–1976; 13,799 seasonal H1N1 1977–2009; 40,643 pandemic H1N1 2009–2013; 1054 H2N2 1957–1968; and 45,010 seasonal H3N2 1968–2013 (Table S2 in File S1). MAFFT, a Multiple Sequence Alignment server was used for the alignment of protein sequences [20].

### Prediction of CD4+ T-cell epitopes using NETMHCIIPAN

A comprehensive evaluation of the Major Histocompatibility Complex (MHC) class II or HLA class II peptide binding prediction servers reported NETMHCIIPAN (epitope binding prediction tool); they based their evaluation in terms of the area under the receiver operating characteristic curve (A_ROC_>0.9) [21]. Hence, NETMHCIIPAN was chosen to calculate the binding affinities of peptide-HLA-DRB1 alleles and to identify the potential CD4+ T-cell epitopes within the H7N9 proteome. NETMHCIIPAN classifies the epitopes as strong binder, weak binder and no binder to selected MHC II alleles based on the binding affinity thresholds ≤50 nM, >50 nM to ≤500 nM and >500 nM, respectively. HLA-DRB1 alleles were selected based on their wide coverage (99%) in the human population [14]. In the current study, we considered only those epitopes predicted to be strong binders for HLA-DRB1 alleles and we disregarded intermediate and weak binders. Identified epitopes were predicted to bind specifically to fourteen HLA-DRB1 alleles: HLA-DRB1*0101, HLA-DRB1*0301, HLA-DRB1*0401, HLA-DRB1*0404, HLA-DRB1*0701, HLA-DRB1*0801, HLA-DRB1*0901, HLA-DRB1*1001, HLA-DRB1*1101, HLA-DRB1*1201, HLA-DRB1*1301, HLA-DRB1*1401, HLA-DRB1*1501 and HLA-DRB1*1601.

### Commonly conserved predicted CD4+ T-cell epitopes

To identify the epitope conservancy, all predicted H7N9 CD4+ T-cell epitopes of each protein were matched against the respective proteins of H1N1 1918–1976, seasonal H1N1 1977–2009, pandemic H1N1 2009–2013, H2N2 1957–1968, and H3N2 1968–2013 viruses using the epitope conservancy analysis tool [22]. The following criteria were applied to select the commonly conserved epitopes: predicted epitopes from the reference strain of H7N9 should be conserved at least in the ≥90% of total sequences of each protein of each subtype and also should have ≥90% amino acid (AA) sequence identity (at least 14 of 15 AAs identical) with that of H1N1 1918–1976, seasonal H1N1 1977–2009, pandemic H1N1 2009–2013, H2N2 1957–1968, and H3N2 1968–2013 [7], [8], [18], [23].

### Conserved and unique predicted CD4+ T-cell epitopes of H7N9 in comparison with human IAVs

In order to acquire unique and conserved epitope datasets, we followed 1) Predicted CD4+ T-cell epitopes of H7N9 are matched with the sequence database of each subtype of IAVs i.e. H1N1 1918–76, seasonal H1N1 1977–2009, pandemic H1N1 2009–2013, H2N2 1957–1968, and H3N2 1968–2013. Epitopes that have ≥90% conservancy are categorized into conserved epitopes of H7N9 with each of the subtype. Epitopes that have <90% conservancy are regarded as epitopes unique to H7N9. Data thus generated is used to calculate whether conserved/unique epitopes in H7N9 are more/less than expected in H7N9 than other strains; 2) Each subtype specific predicted CD4+ T-cell epitopes are matched with the database of H7N9 sequence database. Epitopes that have ≥90% conservancy are categorized into conserved epitopes of specific subtype compared to H7N9. Epitopes that have <90% conservancy are regarded as epitopes unique to specific subtype compared to H7N9. We used two-tailed Chi-square test to compare the observed and expected conserved and unique epitopes.

### Experimental validation of epitopes using the IEDB (Immune Epitope Database)

The predicted CD4+ T-cell epitopes of H7N9 were screened against the IEDB repository, which contains the experimentally defined epitope information on the B-cell, and T-cell of various pathogens present in the published literature [24]. The IEDB contains a total of 5,486 T-cell linear epitopes based on two search criteria: source organism (influenza A virus or influenza virus A); and immune recognition context (T-cell response, MHC binding). A total of 2,659 (∼48%) epitopes of 5,486 induced positive CD4+ and CD8+ T-cell responses in *in-vitro* (with animal and human peripheral blood mononuclear cells (PBMC)) and *in-vivo* (animal models) assays. So, comparing conserved predicted CD4+ T-cell epitopes with the experimentally defined CD4+ T-cell epitope datasets (IEDB) would help in assessing for possible preexisting immunity [6]. First, we conducted predicted CD4+ T-cell epitope sequence homology (≥90%: at least 14 AA of 15 AAs identical) search with the experimentally defined CD4+ T-cell epitope datasets of influenza A viruses collected to identify the experimentally matched predicted CD4+ T-cell epitopes. Similarly, all the CD4+ T-cell epitopes that have ≥90% sequence similarity (9 AA length) with experimentally defined CD8+ T-cell epitopes (from IEDB) were considered as overlapped or nested CD8+ T-cell epitopes.

### Population coverage analysis

Commonly conserved CD4+ T-cell epitopes among avian and human IAVs were screened through a population coverage analysis tool [25] to estimate the population wide coverage in different ethnic populations: Amerindians (Canadian, USA, Mexico), Australian Aborigines, Asian, Arab, Austronesian, Africans, Caucasoid, Hispanic, Mexico Mestizo, Oriental and Polynesian.

## Results

### Surface proteins are distinct than internal proteins

Amino acid (AA) sequences of H7N9 HA and NA shared 39.2% to 47.2% and 41.6% to 45.4% sequence homology, respectively with HA and NA proteins of IAVs (Table 1). When compared with all IAVs used in this study, the internal proteins, PB1, PB2, PA, NP, M1 and NS2 of H7N9 exhibited higher AA sequence homology (93.9% to 99.1%) followed by other internal proteins NS1,M2 (71.3% to 89.6%) and PB1-F2 (62.0% to 74.4%) (Table 1). Higher sequence similarity of internal H7N9 proteins to IAVs proteins suggest that preexisting immunity could be predominantly directed towards these regions.

**Table 1:** Comparison of amino acid sequence identity of 11 protein segments of newly emerged avian influenza (H7N9) viruses in China with H1N1, H2N2, and H3N2 virus subtypes.

Protein Segments	Amino acid sequence identity (%)				
	H1N1 1918–1976	Seasonal H1N1 1977–2009	Pandemic H1N1 2009–2013	H2N2 1957–1968	H3N2 1968–2013
**PB2**	96.0 to 98.1	94.4 to 94.8	94.4 to 97.6	95.2 to 95.6	89.7 to 97.7
**PB1**	95.6 to 97.3	93.9 to 96.0	95.2 to 95.7	96.8 to 97.8	96.5 to 97.4
**PB1-F2**	62.0 to 63.1	34.4 to 36.6^#^	32.2 to 34.3^#^	70.0 to 71.2	62.4 to 74.4
**PA**	95.2 to 96.6	94.5 to 95.8	95.9 to 96.2	94.4 to 95.8	93.2 to 93.7
**HA**	41.7 to 42.7	40.3 to 41.5	39.2 to 41.2	39.2 to 39.9	45.4 to 47.2
**NP**	93.7 to 95.4	91.3 to 91.5	92.1 to 92.8	90.7 to 91.2	91.1 to 92.9
**NA**	42.7 to 43.3	42.0 to 43.1	41.6 to 42.7	44.6 to 45.4	44.1 to 44.8
**M1**	91.6 to 92.0	90.2 to 91.6	91.6 to 92.4	91.4 to 91.6	90.4 to 91.6
**M2**	81.4 to 84.5	79.3 to 80.6	88.7 to 89.6	81.4 to 82.3	81.4 to 84.5
**NS1**	75.9 to 80.8	74.7 to 78.0	75.7 to 76.2	75.9 to 80.3	71.3 to 72.9
**NS2**	91.7 to 95.0	88.4 to 92.5	87.6 to 88.7	92.5 to 93.3	91.7 to 93.7

PB2: RNA polymerase subunit B2; PB1: RNA polymerase subunit B1; PA: RNA polymerase subunit A; HA: hemagglutinin; NP: nucleoprotein; NA: neuraminidase; M1 and M2: matrix proteins; NS1 and NS2: nonstructural protein 1 and 2. ^#^: partial genes available.

### Less conserved and more unique predicted CD4+ T-cell epitopes of H7N9 when compared to human IAVs

Overall, conserved epitopes of internal proteins were less than expected, and unique epitopes of internal proteins were more than expected between H7N9 vs. each of other IAVs. This could be due to the distinct genetic nature of H7N9 when compared to other IAV subtypes. This reasoning is exemplified when H7N9 was compared to the oldest strain i.e. H1N1 1918 (p = 0.0001). For H2N2 and pandemic 2009 H1N1, the results were not significant (p = 0.7242) (Table S3 in File S1). It should be noted that all predicted CD4+ T-cell epitopes are generated in an overlapping fashion from protein sequences. Hence, any change in protein sequence could influence the sequence and the number of predicted CD4+ T-cell epitopes since amino acid change can alter the binding affinity with respective MHC allele.

### Commonly conserved predicted CD4+ T-cell epitopes among avian (H7N9) and human IAVs

**Figure 2:**
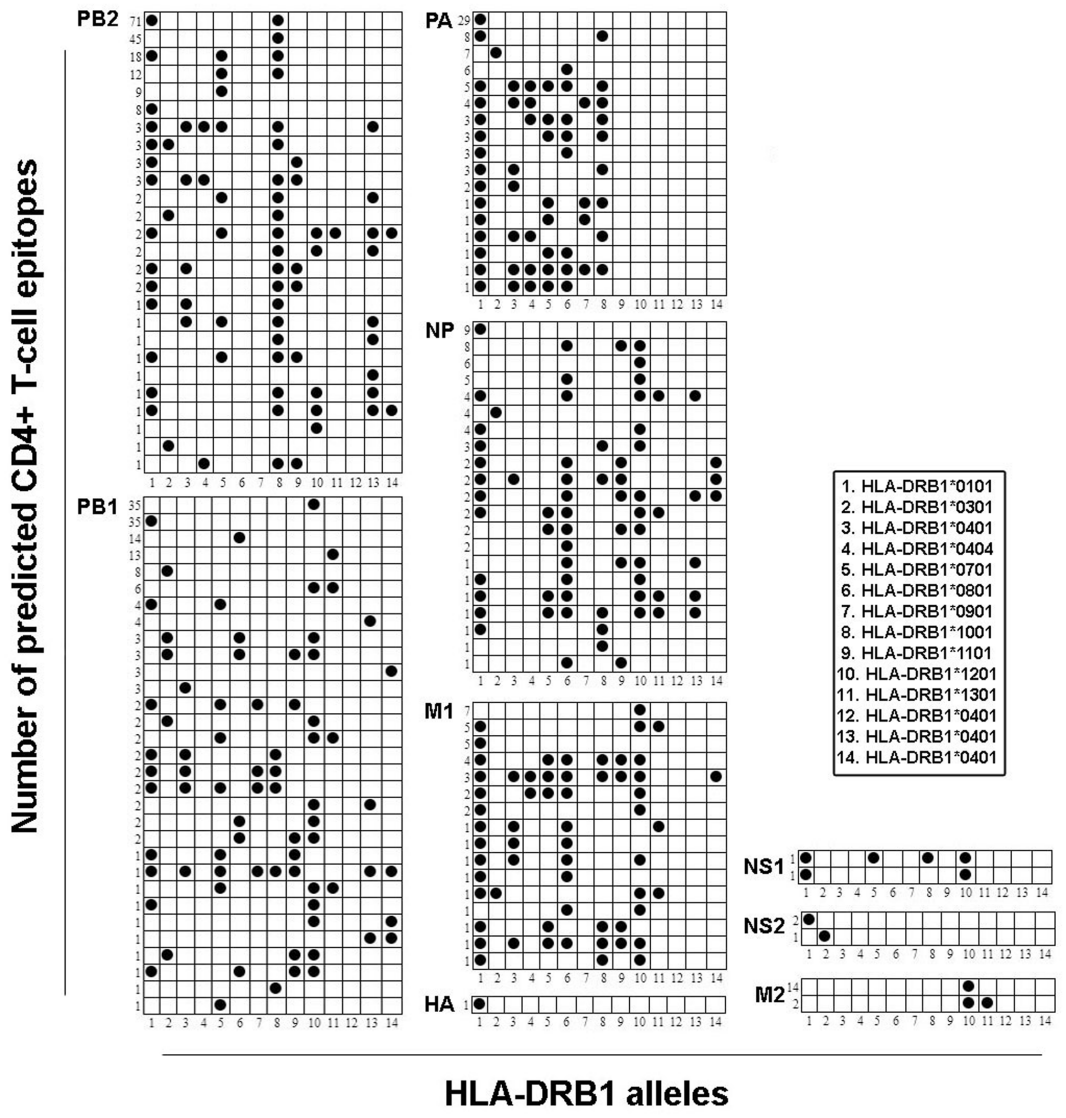
HLA-DRB1 alleles restriction of predicted commonly conserved CD4+ T-cell epitopes. Shown in each panel from A to I are commonly conserved CD4+ T-cell epitopes of nine influenza A virus proteins. Shown on the X-axis are fourteen HLA-DRB1 alleles. On Y-axis are the numbers of predicted CD4+ T-cell epitopes. Each solid circle denotes the HLA-DRB1 allele restriction and promiscuity of identified epitopes.

We conducted in-depth analysis to identify the predicted CD4+ T-cell epitopes of H7N9 that are commonly conserved across all human IAVs and their respective HLA-DRB1 alleles. Table 2 (column 3) contains information on the number of commonly conserved CD4+ T-cell epitopes. Only one (0.86%) of the 116 H7N9 HA predicted CD4+ T-cell epitopes was conserved over the entire evolution of all human IAV viruses (column B of Table 2). This predicted CD4+ T-cell epitope exhibited a strong binding affinity with the HLA-DRB1*0101 (Figure 2, column 2 of Table S4 in File S1). Despite the presence of 118 epitopes in H7N9 NA protein, none were observed to be conserved across all IAVs. The maximum number of epitopes were identified within the PB2 (197/300: 65.7%) followed by PB1 (159/276: 57.6%), PA (79/146: 54.1%), M1 (37/102: 36.3%), and NP (62/178: 35%) and minimum number of epitopes within NS2 (2/43: 4.1%), and NS1 (3/78: 4%) proteins. None of the commonly conserved epitopes were identified within PB1-F2 protein. M2 epitopes (16/32: 50%) are commonly conserved only between H7N9 and 2009 H1N1 and H3N2 viruses. In summary, 556 of 1408 (39.5%) H7N9 predicted CD4+ T-cell epitopes were commonly conserved (≥90%) throughout the evolution of IAV viruses.

**Table 2:** Information CD4+ T-cell epitopes that are commonly conserved between H7N9 viruses and human influenza A virus (IAV) subtypes (H1N1, H2N2, H3N2).

Protein segments	(A) Predicted epitopes within the proteins of H7N9 by NetMHCIIpan tool	(B) Commonly conserved predicted epitopes in IAV and H7N9 sequences (% of conservancy) % of conservancy = B/A*100	Experimentally defined commonly conserved overlapping T-cell epitopes in human and mice studies (% of conservancy) [source: IEDB, immune epitope database]	
			(C) CD4+ T-cell epitopes % of conservancy = C/B*100	(D) CD8+ T-cell epitopes within CD4+ T-cell epitopes % of conservancy = D/C*100
HA	116	1 (0.9)	1 (100.0)	1 (100.0)
NA	118	0	0	0
PB2	300	197 (65.7)	68 (34.5)	11 (16.1)
PB1	276	159 (57.6)	57 (35.8)	20 (35.1)
PB1-F2	19	0	0	0
PA	146	79 (54.1)	32 (40.5)	3 (9.4)
NP	178	62 (35.0)	47 (75.8)	14 (29.7)
NS2	43	2 (4.7)	2 (100.0)	1 (50.0)
NS1	78	3 (3.8)	2 (66.7)	2 (100.0)
M1	102	37 (36.3)	28 (75.6)	5 (18.0)
M2	32	*16 (50.0)*	*16 (100.0)*	*2 (12.5)*
Total	1408	556 (39.5)	253(45.5)	59 (23.3)
Presence of preexisting T-cell immunity in human population against 2013 H7N9 virus is			45.50%	

Italicized numbers refer to conserved regions between H7N9 and each of 2009 H1N1 and H3N2.

Figure 2 (column 2 of Table S4 in File S1) represents the respective predicted HLA-DRB1 allele restriction of commonly conserved predicted CD4+ T-cell epitopes. All predicted PB1 epitopes exhibited strong binding affinity with respective HLA-DRB1 alleles except with HLA-DRB1*404 and HLA-DRB1*1401 alleles. The majority of epitopes (117/159 = 73.5%) were bound with a single DRB1 allele. Forty-two of these 159 (26%) epitopes were noticed to be highly promiscuous with strong binding affinity with more than one HLA-DRB1 allele. Sixty-five of 197 (33%) of PB2 epitopes showed strong binding affinity with a single HLA-DRB1 allele. One hundred and thirty two of 197 (67%) PB2 epitopes were found to be highly promiscuous in nature. Twenty nine of 79 (36.7%) PA epitopes had a strong binding affinity to the single allele HLA-DRB1*0101.Forty eight (77.4%) of 62 NP epitopes bound with more than two HLA-DRB1 alleles. Twenty five of 37 (67.5%) M1 epitopes showed high binding affinity with more than one allele. Two NS2 epitopes exhibited a high binding affinity with HLA-DRB1*0101, HLA-DRB1*0701, HLA-DRB1*1001, and HLA-DRB1*1201. The three predicted NS1 epitopes showed a high binding affinity with the HLA-DRB1*0101 and HLA-DRB1*0301 alleles. Fourteen of 16 (87.5%) M2 epitopes showed higher binding affinity with the HLA-DRB1*1201 allele.

### Immunogenic potential of commonly conserved predicted CD4+ T-cell epitopes

IEDB contains information on experimentally validated B-cell and T-cell epitopes that are published in the literature [6], [24]. Hence, the immunogenic potential of predicted CD4+ T-cell epitopes can be confirmed by screening against the IEDB [6]. Table S4 in File S1 presents this data. All the relevant information of each epitope (its identification number (ID of IEDB), hosts, and MHC II alleles) were tabulated in column 4 of Table S4 in File S1. Overall, 253 of the 556 (45.5%) predicted CD4+ T-cell epitopes are reported to elicit CD4+ T-cell responses with PBMCs and also in animal models (information obtained by screening predicted epitopes with IEDB). Based on screening against IEDB database, the overall preexisting CD4+ T-cell cross-reactivity can be estimated to be 45.5%; suggesting the likely presence of preexisting CD4+ T-cell immunity to H7N9 in the human population due to previous exposures to the different IAV subtypes.

### Commonly conserved CD4+ T-cell epitopes had nested CD8+ T-cell epitopes

A recent study by Quiñones-Parra et al [26], has demonstrated the presence of preexisting CD8+ T-cell immunity to H7N9 virus. Hence, we were interested to investigate nested CD8+ T-cell epitopes in our set of CD4+ T-cell epitopes (as reported in Table S4 in File S1). We further investigated whether any of the nested CD8+ T-cell epitopes identified in our analysis matched with epitopes of NP and M1 that were shown to generate CD8+ T-cell memory responses to H7N9 virus [26].

CD4+ T-cell epitopes that are commonly conserved across IAV and H7N9 were matched with the experimentally defined CD8+ T-cell epitopes of IAVs collected from IEDB. A total of 59 out of 253 (23.3%) experimentally defined CD4+ T-cell epitopes contain CD8+ T-cell epitopes as presented in column 4 (D) of Table 2. All the CD8+ T-cell related information is tabulated in the columns 3 and 4 of Table S4 in File S1. The epitopes that are underlined (column 3 of Table S4 in File S1) induced IFN-γ secretions in *in-vitro* and *in-vivo* experiments based on the IEDB. The last column of Table S4 in File S1 (CD8+ T-cell epitopes assay results) contains the results that were observed in the different experiments based on the IEDB. Information on experimentally verified nested CD8+ T-cell epitopes is tabulated in Table S5 in File S1. Interestingly, we found that many of nested CD8+ T-cell epitopes within our commonly conserved CD4+ T-cell epitopes were shown to generate robust CD8+ T-cell memory responses to H7N9 virus and to human IAVs [26]. All such nested CD8+ T-cell epitopes are bold-faced in Table S5 in File S1.

### Commonly conserved CD4+ T-cell epitopes vary across ethnicities

**Figure 3:**
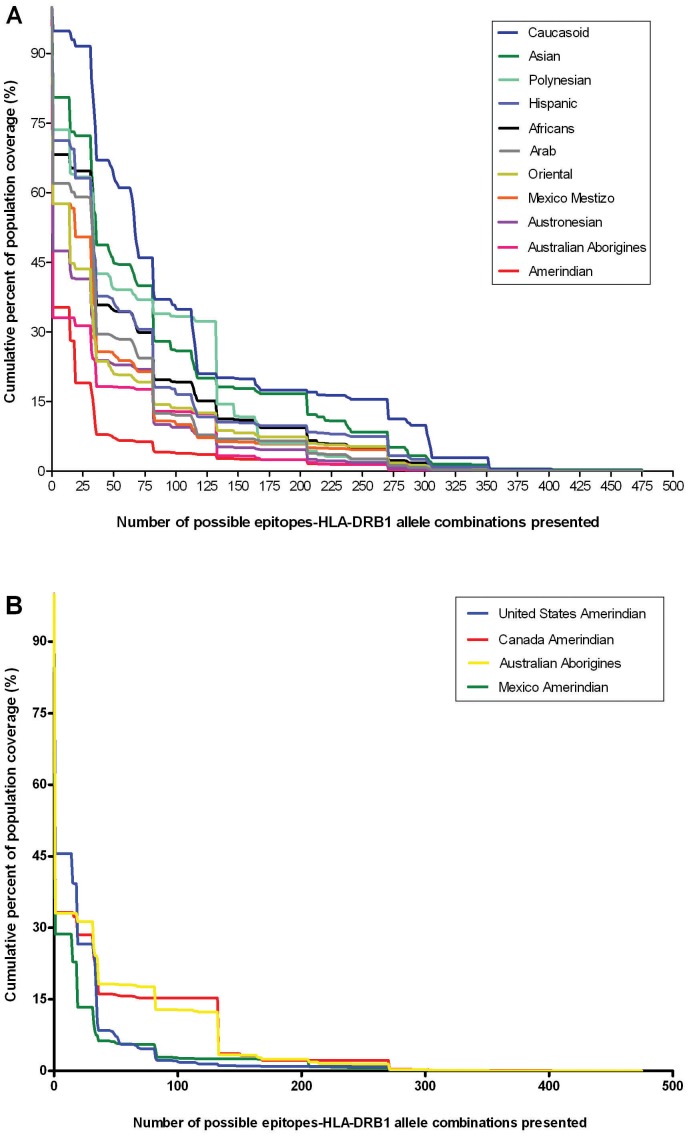
Population coverage analysis of identified commonly conserved CD4+ T-cell epitopes - 3A) all ethnicities groups, and 3B) only indigenous groups. The identified commonly conserved CD4+ T-cell epitopes provide broad population coverage. Based on the binding data for each HLA class II-restricted DRB1 alleles, theoretical population coverage was calculated. The number of possible epitope-HLA allele combinations as a function of the fraction of each ethnic population (%) is shown.

HLA alleles likely bind to highly conserved regions of viral proteins [27]. Hence, prevalence of HLA alleles in population will determine the likely set of peptides (targets) to become T-cell epitopes. This in turn will influence the coverage and/or robustness of T-cell immunity in a population. We have conducted population coverage analysis of commonly conserved CD4+ T-cell epitopes in the context of HLA-DRB1 alleles across different ethnicities. As shown in Figure 3A and 3B, it is evident that the degree of preexisting CD4+ T-cell immunity to H7N9 would vary considerably across different ethnicities with lowest coverage in indigenous or aboriginal or Amerindians populations from Australia (33.08%), Canada (33.23%), Mexico (28.65%), and United States (45.62%) when compared with other ethnicities (57.77% to 94.84%). Given the role of preexisting CD4+ T-cell immunity in limiting disease severity, this ethnic bias would place indigenous population vulnerable to infection in the wake of H7N9 pandemic.

## Discussion

H7N9 remains a global public health concern because of its pandemic potential: its persistent evolution [28], [29]; sporadic human cases [30]; human co-infection with H7N9 and seasonal H3N2 virus [31]; limited knowledge on the source of infection and the reservoirs; and many other uncertain questions [32]. Serological observations reported from the H7N9 outbreak region (Zhejiang province, China) revealed a lack of neutralizing antibodies against H7N9 in the general population (age range 1-88 years) and 6.3% of poultry workers were seropositive with HI titers ≥80 [4]. In the absence of detectable humoral immunity, evidence from human and non-human models demonstrated the protective role of epitope-specific preexisting CD4+ T-cell immunity in attenuating the influenza disease by influencing the transmission dynamics of the pathogen [9], [10]. The effects of preexisting CD4+ T-cell immunity manifest as a prolonged incubation period [33], reduced severity of the disease [34], and reduced infectiousness [34] - as observed during the pandemic 2009 H1N1. Hence, a preexisting CD4+ T-cell pool directed towards commonly conserved epitopes due to previous infections by human IAVs (H1N1 1918–1976, seasonal H1N1 1977–2009, pandemic H1N1 2009–2013, H2N2 1957–1968, and H3N2 1968–2013) - could potentially provide cross-immune protection to the H7N9 virus. Our whole proteome-wide epitope prediction and conservancy analyses found 39.5% (Table 2) predicted commonly conserved CD4+ T-cell epitopes within the internal proteins of human IAVs and avian H7N9 viruses. Our approach of experimental validation with IEDB repository identified 45.5% (253/556) of predicted commonly conserved CD4+ T-cell-epitopes with immunogenic potential. Overall, the level of commonly conserved CD4+ T-cell epitopes in internal proteins of H7N9 virus (i.e. 45.5%) appears to be bit higher than the 41% found for 2009 pandemic H1N1 [6], this lower fraction of difference could be due to the epitope datasets of IAV subtypes used in both studies. Similar to previous findings [35] only one conserved epitope was identified in surface protein, HA of H7N9 virus. The lack of conserved and common CD4+ T-cell epitopes within HA of H7N9 could negatively affect the efficiency of inactivated vaccines [35]; given the synergistic role of antibody and T-cell responses against influenza [36]. In summary, our analyses provide evidence that cross-reactive CD4+ T-cell responses can exist between serologically distinct IAV subtypes and could even provide protective role against unencountered strains, including H7N9 virus [16].

Based on our population coverage analyses, it can be said that preexisting CD4+ T-cell immunity to H7N9 virus varies across different ethnicities especially with lower coverage observed in indigenous population (Figure 3). This could mean that indigenous population may be highly vulnerable to H7N9 infection. This observation gains significance in the wake of recent findings that indigenous population could have diminished preexisting CD8+ T-cell responses to H7N9 virus [26]. Further, our findings are in similar lines with reports of severe illness in indigenous or aboriginal populations of the Canada, United States, Australia, New Zealand, and other parts of Oceania during 1918, 1957 and 2009 H1N1 pandemics [37]–[44]. In Canada, during 2009 H1N1 pandemic indigenous populations were 6.5 times more likely to be admitted to an ICU compared to non- indigenous populations [39], [40]. The reason for this high susceptibility can be attributed to many factors: ethnicity (independently associated with an increased risk of infection), co-morbidities, adverse social determinants of health, limited access to medical care facilities [38], [39] and lack of HLA alleles that present highly conserved epitopes among IAV subtypes [26]. With regards to China where H7N9 is currently restricted, the ethnic (Oriental) population coverage is 55.77% (based on commonly conserved CD4+ T-cell epitopes). Though H7N9 caused severe and fatal illness in different areas of China, small number of cases (4%) are clinically milder suggesting the broad clinical spectrum of H7N9 [45], [46]. Hence, it is possible that differences in clinical spectrum is influenced by preexisting CD4+ T-cell immunity as seen in pandemic 2009 H1N1 [9], [10], [33], [34]. However, this claim remains purely speculative in the absence of experimental investigations towards H7N9. It should also be noted that coverage of commonly conserved CD4+ T cell epitopes in Oriental population (55.77%) is less compared to Caucasoid population (94.84%).

Previous studies have reported, epitopes that can generate both CD4+ and CD8+ T-cell responses due to their sharing of epitope regions are particularly suitable as vaccine antigens and generate robust immune responses [47]–[50]. We have identified CD8+ T-cell epitopes (length 9 AA) that are localized within 23.3% of commonly conserved CD4+ T-cell epitopes (45.5%) (Tables S4 and S5 in File S1). The immunogenic potential of these CD8+ T-cells has also been experimentally proven (as reported in IEDB). Most of our nested CD8+ T-cell epitopes (in CD4+ T-cell epitopes) match with CD8+ T-cell epitopes that were shown to generate recall CD8+ T-cell responses to H7N9 virus by Quiñones-Parra et al. [26]. Given the role of CD4+ T-cell help in the activation and maintenance of CD8+ T-cell effector and memory responses, our study provides evidence that there could be CD4+ T-cell help to generate robust CD8+ T-cell recall responses to H7N9 infection.

Our study has several limitations that should be considered when interpreting findings of our study. Most notably, the binding affinity between epitope-HLA predicts the potential epitope, which is not necessarily reflective of T-cell response. Therefore, T-cell proliferations assays are needed to evaluate the predicted epitopes. Nevertheless, our study provides compelling experimental evidence from published reports and epitope data repository (IEDB). Next, our epitope prediction analysis was restricted to only fourteen HLA-DRB1 alleles - albeit highly prevalent ones - and could be extended to other HLA class II genes: HLA-DRB3, HLA-DRB4, HLA-DRB5, HLA-DP and HLA-DQ. The updated NETMHCIIPAN 3.0 predictor [51] was designed to conduct the computational epitope predictions with all HLA class II genes. Further, comparative immunological and genetic assays using human PBMCs of vulnerable ethnicities (notably indigenous groups) and other ethnic populations are important to understand the genetic reasons behind the high risk of indigenous populations from influenza infection.

To conclude, this study demonstrates the likely evidence for preexisting cross-reactive CD4+ T-cell immunity directed towards commonly conserved epitopes within internal proteins of H7N9 in different ethnicities due to previous exposures to different IAVs either through natural infections or through the seasonal influenza immunizations. The study also provides insights into vulnerability of indigenous population to H7N9 virus in case of H7N9 pandemic. This information is crucial for public health policy people in targeting priority groups for immunization programs. Information on overlapping immunogenic CD4+ and CD8+ T-cell epitopes that are commonly conserved within internal proteins is also useful towards the design of universal vaccines against emerging influenza viruses.

## Supporting Information

File S1 **Supporting Tables.** Table S1. Avian influenza A(H7N9) virus gene segments sequences isolated (from human) in 2013 from China used in the study (collected from GISAID Epiflu Database). Table S2. Protein sequences of human IAV subtypes used in the analysis. Table S3. Conserved and unique predicted CD4+ T-cell epitopes of H7N9 in comparison with human IAVs. Table S4. CD4+ T-cell epitopes that are commonly conserved between avian H7N9 and human IAV subtypes and their experimental verification using IEDB. Table S5. Experimentally defined CD8+ T-cell epitopes nested within commonly conserved CD4+ T-cell epitopes.
(DOC)
