# Supplementary material for: Preexisting CD4+ T-Cell Immunity in Human Population to Avian Influenza H7N9 Virus: Whole Proteome-Wide Immunoinformatics Analyses
Source: PLoS One. 2014 Mar 7;9(3):e91273. doi: 10.1371/journal.pone.0091273 (PMC3946744; doi:10.1371/journal.pone.0091273)
Supplement: File S1 — Supporting Tables. Table S1. Avian influenza A(H7N9) virus gene segments sequences isolated (from human) in 2013 from China used in the study (collected from GISAID Epiflu Database). Table S2. Protein sequences of human IAV subtypes used in the analysis. Table S3. Conserved and unique predicted CD4+ T-cell epitopes of H7N9 in comparison with human IAVs. Table S4. CD4+ T-cell epitopes that are commonly conserved between avian H7N9 and human IAV subtypes and their experimental verification using IEDB. Table S5. Experimentally defined CD8+ T-cell epitopes nested within commonly conserved CD4+ T-cell epitopes. (DOC) [file pone.0091273.s001.doc]

**Supporting Information**

**Preexisting CD4+ T-cell immunity in human population to avian influenza H7N9 virus: whole proteome-wide immunoinformatics analyses**

Venkata R Duvvuri^1,*^, Bhargavi Duvvuri^1^, Christilda Alice^1^, Gillian E Wu^3^,

Jonathan B Gubbay^2,4,5,6^, Jianhong Wu^1,3^

^1^Centre for Disease Modelling, York Institute of Health Research, Toronto, M3J 1P3, Canada.

^2^ The Hospital for Sick Children, Toronto, M5G 0A4, Canada.

^3^ York University, Toronto, M3J 1P3, Canada.

^4^ Public Health Ontario, Toronto, M9P 3T1, Canada.

^5^ University of Toronto, Toronto, M5S 2J7, Canada.

^6^ Mount Sinai Hospital, Toronto, M5G 1X5, Canada.

**Table S1 Page 2**

**Table S2 Page 3**

**Table S3 Page 4**

**Table S4 Pages 5 - 8**

**Table S5 Pages 9 - 10**

**Table S1. Avian influenza A(H7N9) virus gene segments sequences isolated (from human) in 2013 from China used in the study (collected from GISAID Epiflu Database).**

| **Isolate ID** | **Isolate name** | **Collection date** | ***In-vivo* antiviral resistance (genotype)** | **Originating Laboratory** | **Authors/ Submitter** |
| --- | --- | --- | --- | --- | --- |
| EPI_ISL_138737 | A/Shanghai/1/2013 | 2013-02-26 | Adamantines | WHO-CNIC, China | Yang, Lei |
| EPI_ISL_138738 | A/Shanghai/2/2013 | 2013-03-05 | Adamantines | WHO-CNIC, China | Yang, Lei |
| EPI_ISL_138739 | A/Anhui/1/2013 | 2013-03-20 | Adamantines | WHO-CNIC, China | Yang, Lei |
| EPI_ISL_138977 | A/Hangzhou/1/2013 | 2013-03-24 | unknown | Hangzhou CDC | Li, J; Pan, JC; Pu, XY; Yu, XF; Kou, Y; Zhou,YY |

GISAID: Global Initiative on Sharing All Influenza Data.

WHO-CNIC: WHO Chinese National Influenza Center.

**Table S2. Protein sequences of human IAV subtypes used in the analysis.**

| **Influenza A virus** | **Number of Protein sequences collected** | | | | | | | | | | |
| --- | --- | --- | --- | --- | --- | --- | --- | --- | --- | --- | --- |
| **Subtype** | **PB2** | **PB1** | **PB1-F2** | **PA** | **HA** | **NP** | **NA** | **M1** | **M2** | **NS1** | **NS2** |
| H1N1 1918-1976 | 57 | 73 | 51 | 62 | 70 | 74 | 68 | 68 | 66 | 145 | 70 |
| seasonal H1N1 1977-2009 | 979 | 1123 | 1114 | 986 | 1682 | 999 | 1361 | 1973 | 1588 | 901 | 1093 |
| pandemic H1N1 2009-2013 | 4367 | 4321 | 119 | 3463 | 4778 | 3578 | 1727 | 5622 | 5863 | 3443 | 3362 |
| H2N2 1957-1968 | 99 | 65 | 67 | 86 | 70 | 109 | 93 | 124 | 122 | 122 | 97 |
| H3N2 1968-2013 | 3225 | 3618 | 3376 | 3828 | 5910 | 4221 | 2127 | 5499 | 5783 | 4071 | 3352 |
| **Total sequences** | **8727** | **9200** | **4727** | **8425** | **12510** | **8981** | **5376** | **13286** | **13422** | **8682** | **7974** |

**Table S3. Conserved and unique predicted CD4+ T-cell epitopes of H7N9 in comparison with human IAVs.**

|  | **H7N9** | **H1N1 1918** | **Total** |  | **H7N9** | **Seasonal H1N1** | **Total** |
| --- | --- | --- | --- | --- | --- | --- | --- |
| **Conserved** | 703 | 984 | 1687 |  | 728 | 709 | 1437 |
| **Unique** | 705 | 558 | 1263 |  | 680 | 536 | 1216 |
| **Total** | 1408 | 1542 | 2950 |  | 1408 | 1245 | 2653 |
| Chi-square P-value | 0.0001 | | |  | 0.0061 | | |
|  | | | | | | | |
|  | **H7N9** | **Pandemic 2009 H1N1** | **Total** |  | **H7N9** | **H2N2** | **Total** |
| **Conserved** | 881 | 930 | 1811 |  | 900 | 910 | 1810 |
| **Unique** | 527 | 545 | 1072 |  | 508 | 530 | 1038 |
| **Total** | 1408 | 1475 | 2883 |  | 1408 | 1440 | 2848 |
| Chi-square P-value | 0.8126 | | |  | 0.7246 | | |
|  | | | | | | | |
|  | **H7N9** | **H3N2** | **Total** |  |  | | |
| **Conserved** | 743 | 843 | 1586 |  |
| **Unique** | 665 | 627 | 1292 |  |
| **Total** | 1408 | 1470 | 2878 |  |
| Chi-square P-value | 0.015 | | |  |

**Table S4. CD4+ T-cell epitopes that are commonly conserved between avian H7N9 and human IAV subtypes and their experimental verification using IEDB.**

|  |  |  | **Experimentally identified CD4+ T-cell epitopes deposited in IEDB** | | | |
| --- | --- | --- | --- | --- | --- | --- |
| **Epitope position** | **Possible HLA-DRB1 alleles (MHC class II)** | **Overlapping CD4+ T-cell epitopes** | **Epitope ID in IEDB** | **Hosts** | **MHC II alleles determined and test results** | **CD8+ T-cell epitopes assay results [detailed in Table S5]** |
| **Hemagglutinin (HA)** | | | | | | |
| 340-353 | DRB1*0101 | **RGLFGAIAGFIENGW** | 178160 | Human | DRB1*0101 | Positive |
| **Polymerase basic protein 2 (PB2)** | | | | | | |
| 1-25 | DRB1*0404; DRB1*0701; DRB1*1001; DRB1*1101 | MERIKELRDLMSQSRTREILTKTTV |  |  |  | Positive and Negative |
| 39 - 63 | DRB1*0101; DRB1*0401; DRB1*0404; DRB1*1001; DRB1*1101 | QEK**NPALRMKWMMAMKYPITADKRI** | 129189; 129508 | Human | X | Positive and Negative |
| 82 - 101 | DRB1*0101, DRB1*1001 | NDAGSDRVMVSPLAVTWWNR |  |  |  | Negative |
| 129 -149 | DRB1*1001 | **TFGPVHFRNQVKIRRRVDIN**P | 129074; 129664 | Human | X | Negative |
| 158 -172 | DRB1*1001 | EAQDVIMEVVFPNEV |  |  |  | Negative |
| 196 - 231 | DRB1*0101, DRB1*1001 | CKIAPLMVA**YMLERELVRKTRFLPVAGGTSSVYI**EV | 97752; 97779 | Human | X | Positive |
| 234 - 292 | DRB1*0101, DRB1*0701, DRB1*1001, DRB1*1201, DRB1*1301, DRB1*1501, DRB1*1601 | LTQGTCWEQMYTPGGEVRNDDV**DQSLIIAARNIVRRATV**SADPLASLLEMCHSTQIGGV | 128453 | Human, Mouse | X  MHC-II X | Negative |
| 306 - 343 | DRB1*0101, DRB1*0701, DRB1*1001 | QAVDIC**KAAMGLRISSSFSFG**GFTFKRTSGSSVKREEE | 148648 | Human | HLA-DR X | Positive |
| 357 - 387 | DRB1*0101, DRB1*0701, DRB1*1001, DRB1*1101 | HEGYEEFTMVGRR**ATAILRKATRRLIQLI**VS | 129717 | Human | X | Positive |
| 405 - 450 | DRB1*1001, DRB1*0301, DRB1*0701, DRB1*1001 | SQEDCMIKAVRGDLN**FVNRANQRLNPMHQLLRHFQKDAKVLFQNWG** | 97519; 129273; 173065 | Human | X | Negative |
| 495 - 516 | DRB1*0101, DRB1*1001 | VVSIDRFLRVRDQRGNVLLSPE |  |  |  | Positive |
| 521 - 553 | DRB1*0101, DRB1*0701, DRB1*1001 | TQG**TEKLTITYSSSMMWEIN**GPE**SVLVNTYQWI** | 97669; 130059 | Human | X | Positive and Negative |
| 575 - 589 | DRB1*0101, DRB1*1001 | MEFEPFQSLVPKAAR |  |  |  | Negative |
| 592 - 666 | DRB1*0101, DRB1*0401, DRB1*0404, DRB1*0701, DRB1*1001, DRB1*1501 | **YSGFVRVLFQQMRDVLGTFDTVQIIKLLPF**AAAPPKQSRMQFSS**LTVNVRGSGMRI**V**VRGNSPVFNYNK**ATKRLT | 97446; 97502;97656; 97695; 173541 | Human | HLA-DR X | Positive and Negative |
| 704 - 737 | DRB1*1001 | YGPALSINELSNLAKGEKANVLIGQGDVVLVMKR |  |  |  | Negative |
| **Polymerase basic protein 1 (PB1)** | | | | | | |
| 32 - 51 | DRB1*0301, DRB1*0401 | **HGTGTGYTMDTVNRTHKYSE** | 129907; 128843 | Human | X | Positive and Negative |
| 127 - 167 | DRB1*0101, DRB1*0701, DRB1*1201, DRB1*1301, DRB1*1501 | **QTYDWTLNRNQPAATA**LANTIEVFRSNGLTANESGRLIDFL | 129813 | Human | X | Positive and Negative |
| 180 - 196 | DRB1*0801, DRB1*1201 | EITTHFQRKRRVRDNMT |  |  |  |  |
| 213 - 260 | DRB1*0101,DRB1*0801, DRB1*1201, DRB1*1501, DRB1*1601 | NKRSYLIRALTLNTMTKDAERGKLKRRAIATPGMQIRGFVYFVEALAR |  |  |  | Positive and Negative |
| 276 - 297 | DRB1*401, DRB1*0801, DRB1*1201 | NEKKA**KLANVVRKMMTNSQDTE** | 126421 | Human | DRB1*401 | Negative |
| 311 - 360 | DRB1*0101, DRB1*1201, DRB1*1301,DRB1*1501, DRB1*1601 | ENQNP**RMFLAMITYITRNQPEW**FRNV**LSIAPIMFSNKM**ARLGKGYMFESK | 97610; 148595, 148616 | Human | HLA-DR X | Positive and Negative |
| 388 - 421 | DRB1*0101, DRB1*0301, DRB1*1201, DRB1*1301 | KIEKIRPLLIDGTA**SLSPGMMMGMFNMLSTVLGVSILNLGQKK** | 97501; 97653; 97713 | Human Mouse | DRB1*0101;DRB1*0301; DRB1*0401; DRB1*0404; DRB1*0701; DRB1*0901; DRB1*1101; DRB1*1501 | Positive and Negative |
| 441 - 456 | DRB1*0101, DRB1*1001 | LQSSD**DFALIVNAPNH** | 148563 | Human | HLA-DR | Negative |
| 465 - 489 | DRB1*0101, DRB1*0301, DRB1*0801, DRB1*1101, DRB1*1201 | **RFYRTCKLVGINMSKKKSYINRT**GT | 129736; 129104 | Human | X | Positive and Negative |
| 488 - 508 | DRB1*0101, DRB1*0401, DRB1*0701, DRB1*0901, DRB1*1001, DRB1*1201, DRB1*1501, DRB1*1601 | GTFEFTSFFYRYGFVANFS**MELPSFGVSGINE** | 129385 | Human | X | Positive and Negative |
| 522 - 565 | DRB1*0301, DRB1*0701, DRB1*0801, DRB1*1201, DRB1*1301 | **DMSVGVTVIKNNMI**NNDLGPATAQMA**LQLFIKDYRYTYRCHRGD** | 128483; 129308; 128578 | Human Mouse | MHC-II X | Positive and Negative |
| 597 - 612 | DRB1*0701, DRB1*1201, DRB1*1301 | **NMYNIRNLHIPEVC**LK | 148722 | Human | HLA-DR X | Positive and Negative |
| 663 - 685 | DRB1*1201, DRB1*1301 | **THSWIPKRNR**SILNTSQRGILED | 128600 | Human | X | Positive and Negative |
| 691 - 729 | DRB1*0101, DRB1*0301, DRB1*0401, DRB1*0701, DRB1*0801, DRB1*0901, DRB1*1001, DRB1*1101, DRB1*1501, DRB1*1601 | KCCNLFEKFFPSSSYRRPVG**ISSMVEAMVSRARIDARID** | 148645 | Human | HLA-DR X | Positive and Negative |
| **Polymerase (PA)** | | | | | | |
| 1-25 | DRB1*0101, DRB1*0401, DRB1*1001 | MEDFVRQCFNPMIVELAEKAMKEYG |  |  |  |  |
| 441 - 494 | DRB1*0101, DRB1*0401, DRB1*1001 | **MRRNYFTAEVSHCRATEYIMKGVYINTALLNASCAAMDDFQLIPMISKCR TK**EG | 5963, 8306, 14872, 46615, 53218, 97503, 97623; 148589 | Mouse Human | X  HLA-DR X | Positive and Negative |
| 496 - 532 | DRB1*0101, DRB1*0801 | RKTNLYGFIIKGRSHLRNDT**DVVNFVSMEFSLTDPRL** | 128477 | Human | HLA-DR X |  |
| 556 - 597 | DRB1*0101, DRB1*0401, DRB1*0404, DRB1*0701, DRB1*0801, DRB1*1001 | QV**SRPMFLYVRTNGTSKIK**M**KWGMEMRRCLLQSLQQI**ESMIE | 129181, 129991 | Human | X | Negative |
| 629 - 651 | DRB1*0101, DRB1*0401, DRB1*0404, DRB1*0701, DRB1*0801, DRB1*0901, DRB1*1001 | EEGSIGKVCRTLLAKSVFNSLYA |  |  |  | Negative |
| 640 - 678 | DRB1*0101,DRB1*0301, DRB1*0401, DRB1*0404, DRB1*0901, DRB1*1001 | LLAKSVFNSLYASPQLEGFSAESRKLLLIVQALRDNLEP |  |  |  | Positive and Negative |
| 690 - 716 | DRB1*0101, DRB1*0301, DRB1*0701, DRB1*0901, DRB1*1001 | IEECLINDPWVLLNASWFNSFLTHALR |  |  |  |  |
| **Nucleoprotein (NP)** | | | | | | |
| 34 - 52 | DRB1*0101, DRB1*1001, DRB1*1201 | **SGIGRFYI**QMC**TELKLSD** | 97265; 63453 | Human Mouse | X | Positive and Negative |
| 106 - 129 | DRB1*0301, DRB1*0801, DRB1*1201 | **RELILYDKEEIRRIWRQANNGEDA** | 143467; 164290 | Human | DRB1*0301 | Positive |
| 142 - 178 | DRB1*0701, DRB1*0801, DRB1*1001, DRB1*1101, DRB1*1201 | **NLNDATYQRTRALVRTGMDPRMCSLMQGSTLPRRSGA** | 41793; 143309; 49220 | Human | DRB1*0101; DRB1*0701; DRB5*0101 | Positive and Negative |
| 190 - 208 | DRB1*0801, DRB1*1101, DRB1*1201 | **VMELIRMIKRGINDRN**FWR | 5402 | Human | X | Positive and Negative |
| 217 - 236 | DRB1*1201 | **IAYERMCNILKGKFQTAAQR** | 18366; 97416 | Human | HLA-DR1, HLA-DR2 X | Positive and Negative |
| 254 - 277 | DRB1*0101, DRB1*0701, DRB1*0801, DRB1*1001, DRB1*1201, DRB1*1301, DRB1*1501 | EDLIFLA**RSALILRGSVAHKSC**LP | 55796 | Human | X | Positive and Negative |
| 323 - 340 | DRB1*0101, DRB1*0401, DRB1*1201 | **AHKSQLVWMACHSAAFE**D | 126778 | Human | DRB1*0401 | Positive and Negative |
| 378 - 398 | DRB1*0101, DRB1*0801, DRB1*1101, DRB1*1201, DRB1*1501, DRB1*1601 | **TLELRSRYWAIRTRSGGNTNQ** | 35591; 130100 | Human | HLA-DR4 X | Positive and Negative |
| **Matrix protein 1 (M1)** | | | | | | |
| 18 - 33 | DRB1*0101 | G**PLKAEIAQRLEDVFA** | 97554 | Human | HLA-DR1 X | Positive and Negative |
| 42 - 87 | DRB1*0101; DRB1*0401; DRB1*0404; DRB1*0701; DRB1*0801; DRB1*1001; DRB1*1101; DRB1*1201; DRB1*1601 | **LMEWIKTRPILSPLTKGILGFVFTLTVPSER**GLQRRRFVQNALNGN | 97482; 69642 | Human | DRB1*0401; DRB1*1101; DRB1*1201 | Positive and Negative |
| 124 - 139 | DRB1*0101; DRB1*0404; DRB1*1201; DRB1*1301 | **LASCMGLIYNRMGTVT** | 63781 | Human | DRB1*0404 | Positive and Negative |
| 168 - 192 | DRB1*0101; DRB1*0301; DRB1*0401; DRB1*0801; DRB1*1201; DRB1*1301 | T**TNPLIRHENRMVLASTTAKAMEQM** | 65389 | Human | DRB1*0103;DRB1*0401; DRB1*0701;DRB1*1101; DRB1*1501; DRB5*0101 | Positive and Negative |
| **Nonstructural protein 1 (NS1)** | | | | | | |
| 32 - 46 | DRB1*0301 | **FLDRLRRDQKSLRG**R | 127653 | Human | DRB1*0301 | Positive |
| 154-170 | DRB1*0101 | **GAIVG**EISPLPSLPGHT | 149360 | Mouse | HLA-DR1 | Positive |
| **Nonstructural protein 2 (NS2)** | | | | | | |
| 91 - 106 | DRB1*0101,DRB1*0701, DRB1*1001, DRB1*1201 | **ENSFEQITFMQALQ**LL | 13588 | Mouse | X | Positive |
| **Matrix protein 2 (M2)** *– commonly conserved in 2009 H1N1 and H3N2 strains* | | | | | | |
| 24 - 53 | DRB1*1201; DRB1*1301 | **DPLVVAANIIGILHLILWILDRLFFKCIYR** | 26500; 97518 | Mouse Human | X | Positive |

Bold-faced epitopes: experimentally defined CD4+ T-cell epitopes (from IEDB).

Underlined epitopes: positive MHC class I (CD8+ T-cell) epitopes that are experimentally tested in animal models and *in vitro* assays (from IEDB).

X: alleles undetermined.

Empty cells: no information available in the IEDB.

**Table S5. Experimentally defined CD8+ T-cell epitopes nested within commonly conserved CD4+ T-cell epitopes.**

| **Epitope position** | **nested CD8+ T-cell epitopes** | **Epitope ID in IEDB** | **Hosts** | **MHC I alleles determined** |
| --- | --- | --- | --- | --- |
| **Hemagglutinin (HA)** | | | | |
| 340-353 | RGLFGAIAGFIENGW | 178127 | Human | HLA-Class I X |
| **Polymerase basic protein 2 (PB2)** | | | | |
| 14-22 | SRTREILTK | 164387 | Human | HLA-B27 |
| 49-57 | WMMAMKYPI | 72858 | Human | HLA-A*02:01 |
| 196-231 | CKIAPLMVAYMLERELVRKTRFLPVAGGTSSVYIEV | 97752, 97779, 6491 | Human Mouse | HLA-class I X H-2-b class I |
| 322-331 | SFSFGGFTFK | 144475 | Human | HLA-A*03:01, HLA-A*11:01, HLA-A*31:01, HLA-A*33:01, HLA-A*68:01 |
| 359-366 | GYEEFTMV | 23329 | Mouse | H-2-Kb |
| 368-376 | RRATAILRK | 55531 | Mouse | HLA-B*27:05 |
| 500-516 | RFLRVRDQRGNVLLSPE | 129735 | Mouse | HLA-class I |
| 536-553 | MWEINGPESVLVNTYQWI | 129452 | Mouse |  |
| 630-638 | RMQFSSLTV | 54941 | Human | HLA-A*02:01 |
| **Polymerase basic protein 1 (PB1)** | | | | |
| 32-38 | HGTGTGY | 97682 | Human | HLA-class I X |
| 41-49 | DTVNRTHKY | 10514 | Human | HLA-A26 |
| 141-149 | TALANTIEV | 62904 | Mouse | H-2-d class I |
| 215-222 | RSYLIRAL | 56003 | Mouse | H-2-Kb |
| 238-246 | RRAIATPGM | 165648 | Human | HLA-B27 |
| 254-262 | FVEALARSI | 97315 | Human | HLA-A2 |
| 328-344 | NQPEWFRNVLSIAPIMF | 129513 | Human | HLA-class I X |
| 347-360 | KMARLGKGYMF | 32289, 4177 | Human | HLA-B27, HLA-B62 |
| 402-419 | SLSPGMMMGMFNMLSTVLGVSILNLGQ | 37404 | Mouse | HLA-A*02:01 |
| 465 - 489 | RFYRTCKLVGINMSKKKSYINRTGT | 53812, 62591, 144383 | Mouse Human | H-2-Kb, HLA-A*24:01 HLA-A*03:01, HLA-A*11:01, HLA-A*31:01, HLA-A*68:01 |
| 488-503 | GTFEFTSFFYRYGFVANFSME | 97627, 22647 | Human | HLA-A*03:01, HLA-A*11:01, HLA-A*31:01, HLA-A*68:01 |
|  | LPSFGV |  |  |  |
| 528-553 | TVIKNNMINNDLGPATAQM ALQLFIK | 129463, 177335 | Human | HLA-A*02:01, HLA-B7 |
| 597-606 | NMYNIRNLHI | 44947 | Mouse | HLA-A*02:01 |
| 699-722 | FFPSSSYRRPVGISSMVEAMVSRA | 15880, 97781 | Mouse Human | H-2-Kb HLA-Class I X |
| **Polymerase (PA)** | | | | |
| 441-458 | MRRNYFTAEVSHCRATEY | 97503 | Human | HLA- class I X |
| 463-472 | VYINTALLNA | 177367 | Human | HLA-A*02:01 |
| 649-658 | LYASPQLEGF | 176343 | Human | HLA-A*24:02 |
| **Nucleoprotein (NP)** | | | | |
| **32-52** | GR**FYIQMCTEL**KLSDY | 18405, 7136, 97496 | Mouse Human | H-2-d class I HLA-A1 |
| 110-118 | YDKEEIRRI | 175667 | Chicken | BF4 |
| **142 - 178** | NLNDA**TYQRTRALVRTGMDPRM**CSLMQGSTLPRRSGA | 145808, 146073, 49219, 65994 | Human Mouse | HLA-B*15:01 H-2-d class I |
| **190 - 208** | VMELIRMIK**RGINDRNFW**R | 42975, 173003 | Mouse Human | HLA-A11 HLA- class I X |
| **217-235** | IA**YERMCNILKG**KFQTAA | 145824 | Human | HLA-B*15:01 |
| 254-275 | EDL**IFLARSALILRGSVAHKS**C | 9124 | Mouse | HLA-A*02:01 |
| 323-336 | QLVWMACHSAAFED | 97583, 97764 | Human | HLA-A2 |
| 379-396 | LELRSRYWAIRTRSGGNT | 145868 | Human | HLA-B*15:01 |
| **Matrix protein 1 (M1)** | | | | |
| 18-31 | GPLKAEIAQRLEDV | 58177 | Human | HLA-A*02:01 |
| 42-69 | LMEWI**KTRPILSPLTKGILGFVFTLTVP** | 9054, 20356 | Mouse Human | HLA-A*02:01 HLA-A2, HLA-A69 |
| 125-134 | **ASCMGLIY**NR | 144210 | Human | HLA-A*03:01,HLA-A*11:01, HLA-A*31:01, HLA-A*33:01, HLA-A*68:01 |
| 173-181 | **IRHENRMVL** | 28309 | Human | HLA-B39 |
| 179-187 | **MVLASTTAK** | 42959 | Mouse | HLA-A11 |
| **Nonstructural protein 1 (NS1)** | | | | |
| 32-40 | FLDRLRRDQ | 103050 | Mouse | MHC-I X |
| 158-166 | GEISPLPSL | 19312 | Human | HLA-B44 |
| **Nonstructural protein 2 (NS2)** | | | | |
| 97-106 | ITFMQALQLL | 97405 | Human | HLA-A2 |
| **Matrix protein 2 (M2)** | | | | |
| 32-55 | IIGILHLILWILDRLF | 26500, 153854 | Mouse | HLA-A*02:01, HLA-A*24:02 |
| 45-54 | RLFFKCIYR | 144461 | Human | HLA-A*03:01, HLA-A*11:01, HLA-A*31:01, HLA-A*33:01, HLA-A*68:01 |

Bold-faced CD8+ T-cell epitopes were shown to induce recall CD8+ T-cell responses [26].

X: alleles undetermined.

Empty cells: no information available in the IEDB.
